# Redox Mechanism in Na-Ion Battery Cathodes Probed by Advanced Soft X-Ray Spectroscopy

**DOI:** 10.3389/fchem.2020.00816

**Published:** 2020-09-15

**Authors:** Jinpeng Wu, Zhi-xun Shen, Wanli Yang

**Affiliations:** ^1^Geballe Laboratory for Advanced Materials, Stanford University, Stanford, CA, United States; ^2^Advanced Light Source, Lawrence Berkeley National Laboratory, Berkeley, CA, United States; ^3^Stanford Institute for Materials and Energy Sciences, SLAC National Accelerator Laboratory, Menlo Park, CA, United States; ^4^Department of Physics and Applied Physics, Stanford University, Stanford, CA, United States

**Keywords:** Na-ion battery, cathode, redox mechanism, soft X-ray spectroscopy, resonant inelastic X-ray scattering, X-ray absorption spectroscopy

## Abstract

A Na-ion battery (NIB) device is a promising solution for mid-/large-scale energy storage, with the advantages of material abundance, low cost, and environmental benignity. To improve the NIB capacity and retainability, extensive efforts have been put into the developments of NIB electrode materials. The redox activities of the transition metal (TM)-based NIB electrodes are critical in defining the capacity and stability. Here, we provide a comprehensive review on recent studies of the redox mechanisms of NIB cathodes through synchrotron-based soft X-ray absorption spectroscopy (sXAS) and mapping of resonant inelastic X-ray scattering (mRIXS). These soft X-ray techniques are direct and effective tools to fingerprint the TM-3*d* and O-*p* states with both bulk and surface sensitivities. Particularly, 3*d* TM *L*-edge sXAS has been used to quantify the cationic redox contributions to the electrochemical property; however, it suffers from lineshape distortion for the bulk sensitive signals in some scenarios. With the new dimension of information along the emitted photon energy, mRIXS can address the distortion issue of in TM-*L* sXAS; moreover, it also breaks through the limitation of conventional sXAS on detecting unconventional TM and O states, e.g., Mn(I) in NIB anode and oxidized oxygen in NIB cathodes. The mRIXS fingerprint of the oxidized oxygen state enables the detection of the reversibility of the oxygen redox reaction through the evolution of feature intensity upon electrochemical cycling and thus clarifies various misunderstandings in our conventional wisdom. We conclude that, with mRIXS established as a powerful tool, its potential and power will continue to be explored for characterizing novel chemical states in NIB electrodes.

## Introduction

With the increasing penetration of the wind and solar energy into the power grid, large-scale energy storage devices are of severe demand to meet the intermittency of the renewable energy sources. Among the various possible methods, electric energy storage through electrochemical devices, i.e., batteries, have attracted lots of attentions from academia to industry, due to its high flexibility and efficiency (Dunn et al., 2011; Goodenough, 2015). Though the Li-ion battery (LIB) is ubiquitous in the fields of electric vehicles and portable electric devices, and there are more and more concerns on its reserves, cost, and the distribution territory (Whittingham, 2014). The Na-ion battery (NIB) is thus considered as another promising candidate for the next-generation energy storage, especially for the large-scale energy storage, due to its advantages of material abundance, low cost, and environmental benignity (Hwang et al., 2017). Compared with Li^+^, Na^+^ has heavier atomic weight and lower negative potential, leading to inferior capacity and energy density; moreover, the larger ionic radius of Na^+^ also makes the NIB electrodes suffer from sluggish reaction kinetics and vulnerable structural stability, which results in poor rate behavior and deficient cyclability, respectively. On capacity and energy density, the cathode materials generally exhibit specific capacity of 120~280 mA·h·g^−1^ (Xu et al., 2018; Chen et al., 2019; Song et al., 2019), much less than that of the anode materials that are also under scrutiny (Li et al., 2018), indicating that the cathode is the bottleneck of the storage capacity of the NIBs. Therefore, breakthroughs on NIB electrodes are critical challenges for improving the NIB systems, which require both practical optimizations and conceptual innovations based on fundamental understandings.

During electrochemical Na^+^ (de)intercalation process, one or more elements in cathode compounds could be oxidized and reduced, i.e., the redox-active elements or redox centers. While electrochemical and structural characterizations have been extensively conducted, direct characterizations of the redox activities with elemental and chemical sensitivity are essential. In the conventional oxide-based cathode system, 3*d* transition metals (TMs) are the redox centers (Hwang et al., 2017; Chen et al., 2019). The TM redox activities on the surface and in the bulk usually differ in the NIB cathodes and define the capacity and other electrochemical properties together. Various strategies including doping modification, surface treatment, and composite construction have been extensively employed to tune and optimize the TM redox in NIB cathodes (Xiang et al., 2015; Fang et al., 2016; Li et al., 2017; Chen et al., 2019). Currently, detecting, understanding, and tuning the TM redox reactions remain an active topic in research and development of NIBs.

The oxygen (O) activities have attracted a lot of attentions in the oxide-based cathode materials in the past few years. Conventional wisdom often considers O activities as irreversible reactions and are detrimental to the battery performance (Goodenough, 2015). However, recent findings have suggested that O redox reactions could be highly reversible in NIBs. Such a reversible anionic redox, firstly reported in the Li-rich compounds (Sathiya et al., 2013b), has been found in various NIB cathode materials (Xu et al., 2014; Yabuuchi et al., 2014; Du et al., 2016; Mortemard de Boisse et al., 2016; Perez et al., 2016; Rong et al., 2018b; Dai et al., 2019; Wu et al., 2020b). This provides a unique opportunity for improving the energy density and capacity of NIB systems, provided the O redox reactions could be optimized to be highly reversible.

Technically, deciphering the redox mechanisms of the NIB cathodes during the electrochemical cycling requires an incisive probe of the chemical states with elemental sensitivity, e.g., TMs for cationic redox and/or O for anionic redox. X-ray-based characterization techniques, including soft X-ray spectroscopy (SXS) and hard X-ray spectroscopy (HXS), have been widely employed. Compared with HXS, SXS utilizes the relatively low-energy X-ray from about tens eV to about 1.5 keV, covering the low-*Z* ligand *K*-edge and 3*d* TM *L*-edge, which corresponds directly to the *p* states of the ligand element, e.g., O, and *3d* states of the 3*d* TMs, respectively (Lin et al., 2017). Among various SXS techniques, soft X-ray absorption spectroscopy (sXAS), corresponding to the unoccupied conduction-band states with the core-hole existence, has been widely utilized (Olalde-Velasco et al., 2011; Lin et al., 2017). Especially, 3*d* TM-*L* sXAS provides supreme sensitivity to the 3*d* valence states and allows for quantitative analysis of the valence variation upon cycling (Yang et al., 2013; Li et al., 2016). However, such a quantitative probe is limited to surface signal analysis due to the severe lineshape distortion of the spectra collected with deeper probe depth through the so-called fluorescence yield (FY) channel (Achkar et al., 2011). Additionally, some unconventional states that are triggered by electrochemical cycling cannot be sensed through conventional sXAS, as elaborated in this review through several examples of both TMs and O. In particular, we have clarified that sXAS is not conclusive for characterizing the O redox states because the intensity and the lineshape of the O-*K* sXAS are both dominated by TM characters through strong hybridization effects (Yang and Devereaux, 2018; Zhuo et al., 2019; Roychoudhury et al., 2020). In order to tackle these technical limitations of sXAS in both its FY lineshape distortion and its lack of chemical sensitivity, high-efficiency mapping of resonant inelastic X-ray scattering (mRIXS) has been developed. Compared with sXAS, mRIXS reveals a completely new dimension of information along the emitted photon energy (Yang and Devereaux, 2018). By this time, mRIXS has been demonstrated as a powerful tool to probe the redox states in the bulk electrodes for both TMs and O in the NIB cathodes.

Here, in this review, we summarize the recent advancements in SXS for characterizing the redox mechanisms in the NIB cathode materials. Firstly, we introduce several X-ray characterization techniques, especially sXAS and mRIXS, with several other techniques for redox mechanism studies. Secondly, we discuss several representative examples of detecting the 3*d* TM redox mechanism of the NIB cathode materials. We demonstrate the capability of SXS on quantifying the 3*d* TM electronic and chemical states. These examples cover different aspects of TM redox reaction studies on the electrode surface through sXAS, in the bulk through mRIXS, and on novel chemical states through mRIXS. Thirdly, we summarize the recent findings of the O redox reactions in the NIB cathode materials through O-*K* mRIXS. We focus on several critical issues regarding the NIB systems with O redox, such as reversibility and cyclability, performance decay, voltage hysteresis, and kinetics. Fourthly, we provide our perspectives on the future developments of both the spectroscopic techniques and redox mechanisms in the NIB cathode materials. It is important to note that this review is not a general review of NIB materials; instead, it is a focused topical review on characterizations of the challenging and critical redox reactions through sXAS and mRIXS, with the emphasis on O redox reactions of NIB cathodes probed by mRIXS.

## Characterizations of Redox Reactions in Sodium-Ion Battery

In this section, we summarize various characterization techniques that have been prevailingly employed in NIB redox investigations. The synchrotron-based soft X-ray spectroscopies including sXAS and mRIXS are emphasized here. However, structural probes, such as transmission electron microscopy (TEM) and pair distribution function (PDF), and other spectroscopic tools, such as Raman and X-ray photoelectron spectroscopy (XPS), are briefly introduced too due to their popularity. Nonetheless, we note again that the 3*d* TM*-L* and O*-K* sXAS and mRIXS correspond to the spectroscopic excitations to the TM*-3d* and O*-2p* valence states directly, providing the most direct information on their chemical states.

### Transmission Electron Microscopy and Pair Distribution Function

TEM is a powerful technique for characterizing the morphology and structure of the electrode materials in a visualized pattern at atomic lever. Two modes are commonly applied, including scanning TEM (STEM) and selected area electron diffraction (SAED). Several representative applications of TEM in the O redox studies are given here. McCalla *et al*. utilize STEM in a Li-rich model system Li_2_Ir_1−x_Sn_*x*_O_3_ and claim that the observed shortened O–O pairing (about ~2.5 Å), namely, peroxo-like dimers, is responsible for the capacity gain in Li-rich layered electrode materials (McCalla et al., 2015). Similar findings of the shorter O–O separations are also demonstrated in a Na-rich system β-Na_1.7_IrO_3_ (Pearce et al., 2018), but it is indeed controversial to conclude the oxidized O species as the peroxide O–O dimer (Hong et al., 2019). Li *et al*. employ STEM to confirm the native vacancy in the Mn–O slabs and the Na ion in the layer between Mn–O slabs and further clarify that no Mn-ion migration or surface reconstruction occurs (Li et al., 2019b). Moreover, Boisse *et al*. utilize SAED on Na_2_RuO_3_, and they verify the polymorphs of with ordered and disordered honeycomb-type patterns in the TM slabs (Mortemard de Boisse et al., 2016). Susanto *et al*. achieve the SAED pattern of NaFeO_2_ cathode, and they reveal Fe_3_O_4_ formation with O_2_ gas release on the surface, which explains the irreversibility of the NIB cathode material (Susanto et al., 2019).

PDF is a real-space-based technique that utilizes both the Bragg diffraction and the diffuse scattering signals and therefore has an ultra-high sensitivity to short-range ordering and local distortion. With different incident sources, X-ray PDF (xPDF) is sensitive to heavier elements such as TMs, and neutron PDF (nPDF) is sensitive to lighter elements including C, N, O, and F. Rong *et al*. employ both the xPDF and nPDF on the NIB cathode material P3-type Na_0.6_[Li_0.2_Mn_0.8_]O_2_ (Rong et al., 2018b). xPDF reveals the information of the first neighbor Mn–O and Mn–Mn for both the pristine and charged electrodes, and it confirms that the Na^+^ extraction does not incur significant changes on TM framework. nPDF demonstrates the neighboring information of the Na–O and O–O for both the pristine and charged electrodes, and it verifies that the distance of O–O pair changes from 2.637 to 2.506 Å, other than 1.5 Å, indicating very limited O–O peroxide dimers. These findings are consistent with those in other studies (McCalla et al., 2015; Hong et al., 2019). A similar study is also performed in another NIB cathode material P2-Na_0.72_[Li_0.24_Mn_0.76_]O_2_, demonstrating similar findings by PDF technique (Rong et al., 2018a).

Both TEM and PDF provide critical structural information that is associated with the redox mechanism. They are often used as standard probes of battery materials, compensating other spectroscopic experiments on more directly detections of the chemical state evolution.

### Raman Spectroscopy

Raman spectroscopy is employed to characterize the molecule bonding in the materials through detecting vibration and rotation features in a molecule. Several researches utilize this technique to reveal the abovementioned peroxo-like or superoxide-like O species in both LIB and NIB cathode materials. Zhang *et al*. perform *in situ* Raman spectroscopy on Na-rich NIB cathode material Na_1.2_Mn_0.4_Ir_0.4_O_2_, and they claim the formation of peroxo-like O–O dimer and the participation of O redox upon cycling; moreover, the new appeared peak at about 1,109 cm^−1^ is attributed to the superoxide species, which is assumed as the reason of the oxygen loss and capacity fading (Zhang et al., 2019). The study based on Raman spectroscopy is also performed on the Li-rich LIB cathode material Li_2_Ni_1/3_Ru_2/3_O_3_, and similar findings are achieved (Li et al., 2019a). Although not as a direct chemical probe, Raman spectroscopy has provided valuable information on characteristic chemical bonds associated with redox reactions, especially in the alkali-rich systems that likely involve specific peroxo-like species in the charged states.

### X-Ray Photoelectron Spectroscopy

XPS is a photoelectron spectroscopic technique in surface analysis. The inelastic mean free path of electrons is usually up to several nm, resulting in an extreme surface sensitivity. For measuring the surface and interface in battery systems, the probe depth could be tuned within a certain range by employing different incident photon energies (Malmgren et al., 2013). XPS has been widely utilized in the studies of battery redox mechanisms (Philippe et al., 2015; Lin et al., 2017). Especially, many XPS studies found oxidized O species (O^−^/${\text{O}}_{2}^{-}$) in charged battery cathodes that have been widely considered as evidences of lattice O redox (Sathiya et al., 2013a; McCalla et al., 2015; Perez et al., 2016; Yabuuchi et al., 2016; Assat et al., 2017, 2018; Rong et al., 2018b). However, recent clarification shows that, even with hard X-rays, XPS remains unreliable to fingerprint the O redox states in battery materials (Lebens-Higgins et al., 2020).

### X-Ray Absorption Spectroscopy

With incident hard X-ray photons, hard XAS (hXAS) employs X-ray photon energy with few keV to tens of keV, covering the TM 1*s* core level electron excitation. Most importantly, due to the photons with high energy, hard X-ray benefits from deep probe depth with *mm*. hXAS is often performed under *in situ*/*operando* conditions these days due to its advantages in penetration depth and no requirement on high vacuum environment for experiments. A hXAS spectrum is usually divided into two parts, the X-ray near-edge structure (XANES) referring to small energy range (30–50 eV) near absorption edge and the extended X-ray absorption fine structure (EXAFS) referring to large energy range (hundreds eV) above absorption edge. EXAFS is the technique of choice for studying the local structures (e.g., the bond length and coordination numbers) of electrode materials with low crystallinity, or even those amorphous battery materials, both in the synthetic state and after long cycles (Lin et al., 2017; Schoch et al., 2019). The XANES measurement has been intensively used to determine the valence states of a specific TM element, by comparing the edge energy position with that of the reference spectrum (Deb et al., 2005; Yoon et al., 2005; Buchholz et al., 2015). It is also worth noting that 1*s* to 3*d* quadrupole transitions could be observed in 3*d* TM-*K* hXAS as pre-edge features. These pre-edge features are formally dipole-forbidden but quadrupole-allowed, whose intensity is pretty weak. hXAS is much more popular in battery material characterizations, compared with sXAS, due to its advantages in penetration depth, operando conditions, and sample handling (Lin et al., 2017). One should be cautious with the main peak and edge analysis on certain TM elements, e.g., Mn, because careful studies have shown that ligand effect on exactly the same TM oxidation states could lead to dramatically different main peak/edge features (Manceau et al., 2012).

**Figure 1:**
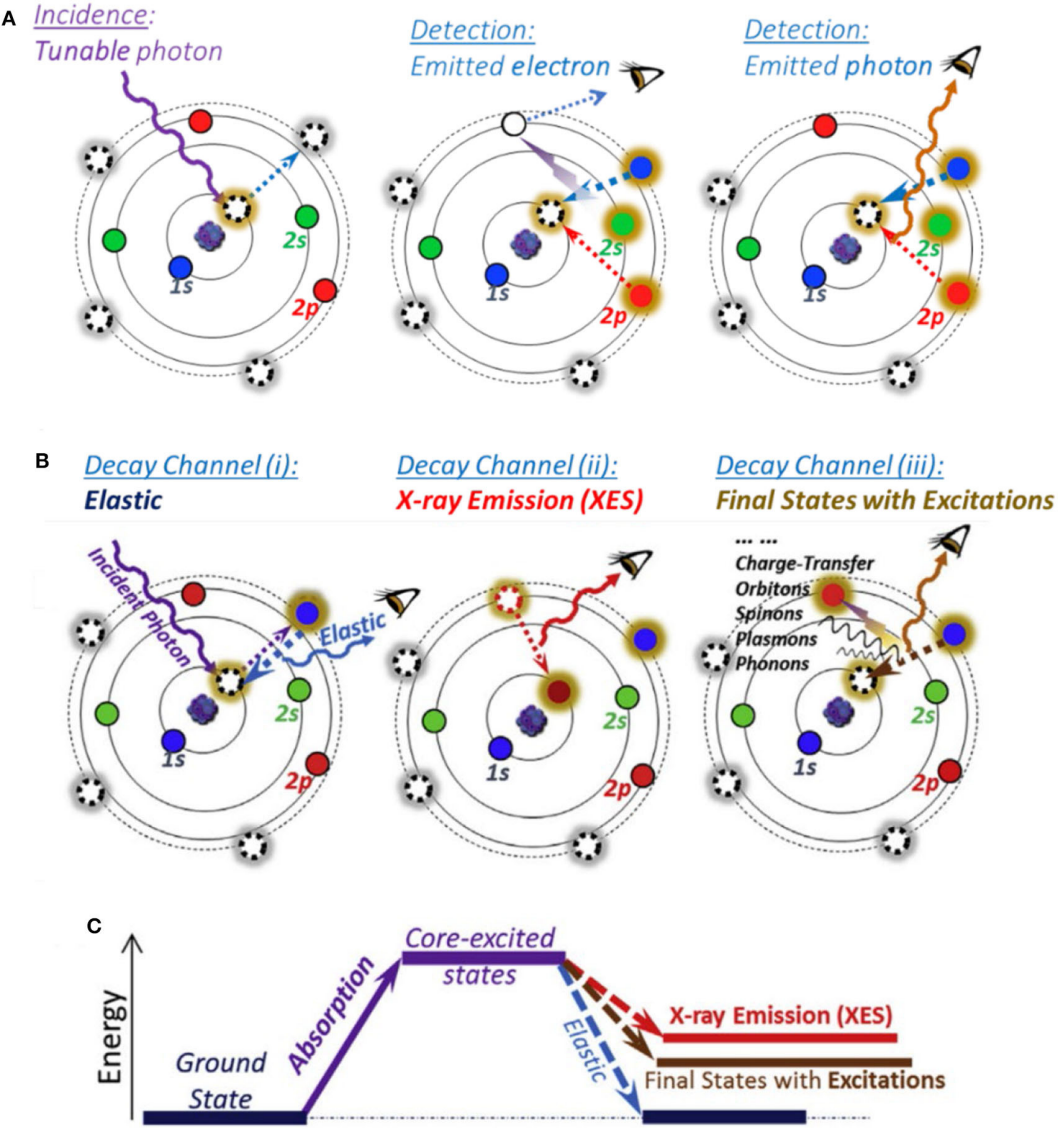
Atomic schematic of soft X-ray spectroscopy (SXS). **(A)** sXAS excitation and decay processes with electron and photon detection modes. **(B)** Different decay channels leading to various features generated on mapping of resonant inelastic X-ray scattering (mRIXS). **(C)** Three types of final states. Reprinted from Yang et al. (2013) and Yang and Devereaux (2018), with permission respectively from Elsevier.

sXAS uses the incident photon energy from tens eV to about 1.5 keV, covering various core level electron excitations of the low-*Z* elements (C, N, and O), *K*-edge, and 3*d* TM-*L* edge (Yang et al., 2013; Yang and Devereaux, 2018). As shown in Figure 1A, sXAS process starts with the absorption of tunable incident photons to excite the core electrons to an unoccupied state (left panel); subsequently, the excited state would decay to fill the core hole, and both electrons and photons would be yielded during the decay process, leading to two kinds of detection modes, namely, total electron yield (TEY; middle panel) and total FY (TFY, right panel). These two modes have different probe depths, i.e., several nm for TEY and 100–200 nm for TFY. sXAS has been well demonstrated as a powerful technique to probe the electronic and chemical states (e.g., formal valence, spin state, and chemical bond configuration) for both TMs and O (Qiao et al., 2012; Li et al., 2016). Compared with 3*d* TM-*K* hXAS, 3*d* TM-*L* sXAS is much more sensitive to the 3*d* TM electronic and chemical states, due to strong 2*p*-3*d* dipole-allowed excitation features. This allows quantitative or quasi-quantitative analysis of the TM redox in the battery electrodes.

However, sXAS often encounters lineshape distortion issues in its bulk sensitive TFY mode. For example, Mn-*L* sXAS-TFY always displays a seriously distorted lineshape, which hinders the reliable quantifications of the bulk signals (Achkar et al., 2011; Qiao et al., 2017). Moreover, due to the lack of capability for differentiating the emitted photons in sXAS FY mode, sXAS sometimes lacks the chemical sensitivity for detecting the unconventional states, such as novel TM states and non-divalent O states, as elaborated in this review.

### Mapping of Resonant Inelastic X-Ray Scattering

To overcome the limitations of sXAS as mentioned above, high-efficiency mRIXS was developed, which could quickly scan across the sXAS edge range with a large energy window of emitted photon energy covering the full soft x-ray range (Qiao et al., 2017; Yang and Devereaux, 2018). Besides, RIXS is a photon-in–photon-out probe with a probe depth of 100–200 nm. In principle, after a core electron is excited by a tunable incident X-ray, the excitation state will then decay to fill the generated core hole through different channels and hence lead to various features, as shown in Figure 1B (Yang and Devereaux, 2018): (i) (left panel) if excitation state decays back to the same ground state, a photon with the same energy with incident X-ray will be released, leading to an elastic feature; (ii) (middle panel) if the core electron is excited to the high-energy continuum far above the absorption threshold, the core hole will be filled by a valence-band electron, leading to the emitted photon energy pinned by the energy gap between the valence electron and the core hole, namely, normal X-ray emission spectroscopy (XES); (iii) (right panel) else, the core hole in the intermediate state will exert a strong influence on the outer shell electrons, leading to various other excitations, including atomic vibrations (phonons), spin flips (spinons or magnons), and charge transfer excitations. Therefore, via the excitation and decay process, the system will get to three types of final states as shown in Figure 1C.

An important application of TM-*L* mRIXS for TM redox mechanism characterization is through the inverse partial FY (iPFY) analysis, which is proved to be a non-distorted bulk-sensitive absorption profile of 3*d* TM-*L* states (Achkar et al., 2011) and has been utilized in several battery systems (to be detailed in *Fingerprint of Bulk Mn Redox in Na*_0.6_*Li*_0.2_*Mn*_0.8_*O*_2_ *by Transition Metal-L Mapping of Resonant Inelastic X-Ray Scattering*) (Dai et al., 2019; Ji et al., 2020; Lee et al., 2020; Wu et al., 2020a,b). Meanwhile, Mn-*L* mRIXS is utilized to detect the novel electronic states of the monovalent Mn in battery electrodes, which cannot be revealed by Mn-*L* sXAS (to be detailed in *Novel Mn(I) State in Na*_*x*_*Mn[Mn*^*II*^*(CN)*_6_*]*_0.81_) (Firouzi et al., 2018). For O redox, O-*K* mRIXS becomes critical and can successfully differentiate the oxidized O feature from the dominating TM-O hybridization signals, which are entangled together in the O-*K* sXAS-TFY profile. At this time, mRIXS has been recognized as the “tool-of-choice” for characterizing O redox reactions in battery electrodes (to be detailed in *Detecting Lattice O Redox States Through Mapping of Resonant Inelastic X-Ray Scattering*) (Yang and Devereaux, 2018).

## Transition Metal Redox in Sodium-Ion Battery Electrodes

3*d* TMs are the most common and conventional redox centers in the NIB electrode materials. Benefiting from the sensitivity of the TM 3*d* states and sharp features from multiple effects, 3*d* TM-*L* SXS is the most direct and reliable probe of the 3*d* TM redox in NIB electrodes (Yang et al., 2013; Li et al., 2016). In this session, we summarize four representative cases. By the Na_0.44_MnO_2_ and Na_*x*_MnFe(CN)_6_·*z*H_2_O systems, we demonstrate the advantages of 3*d* TM-*L* sXAS on quantifying the TM redox activities both on the surface and in the bulk, and we illustrate how the electrochemical performances of the NIB electrodes are determined; and by the Na_0.6_Li_0.2_Mn_0.8_O_2_ and Na_*x*_Mn[Mn(CN)_6_]·2.1H_2_O systems, we showcase the limitations of sXAS and clarify the superiority of TM-*L* mRIXS on providing non-distorted bulk probes and unveiling novel redox couples.

### Surface Mn Redox of Na_0.44_MnO_2_

**Figure 2:**
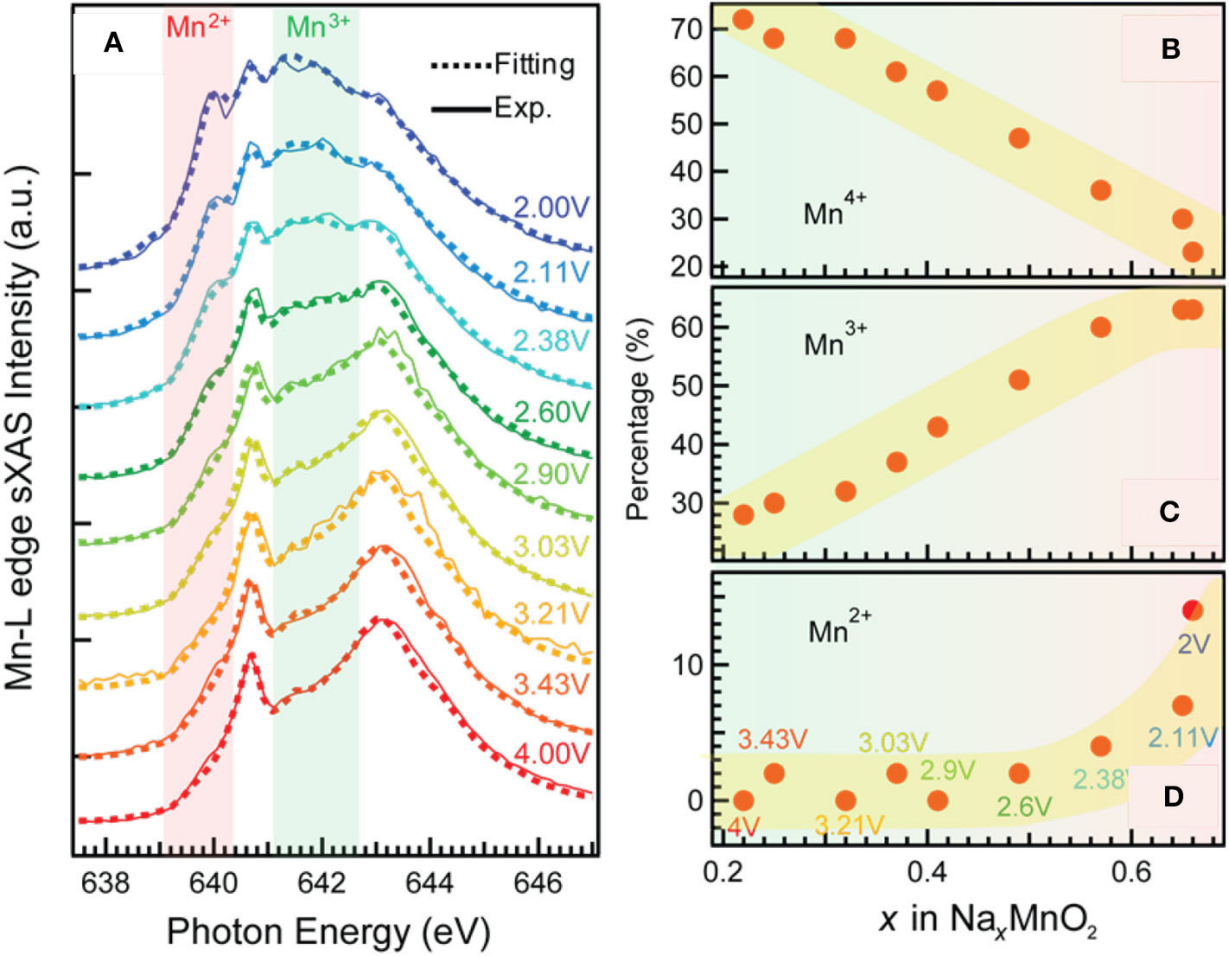
Mn-*L* soft X-ray absorption spectroscopy–total electron yield (sXAS-TEY) spectra collected on a series Na_*x*_MnO_2_ electrodes at various state of charge **(A)** and the variation of Mn^2+/3+/4+^ concentrations **(B–D)** achieved by linear combination of reference spectra of Mn^2+^ (MnO), Mn^3+^ (Mn_2_O_3_), and Mn^4+^ (Li_2_MnO_3_). Reprinted from Qiao et al. (2015a) with permission from Elsevier.

The TM redox reactions on electrode surface usually exert considerable influence on the electrochemical performance of a battery. Mn-based oxide Na_0.44_MnO_2_ is a promising NIB cathode material (Dai et al., 2015). It has the wide and stable tunnel structure, which is suitable for fast and substantial sodium (de)intercalation; it is also applicable in aqueous electrolyte, leading to cost reduction and safety improvement. However, its cycling performance still needs more improvements. Qiao et al. take a scrutiny on this material, and they reveal the mechanisms for the capacity decay by utilizing a comprehensive and quantitative sXAS analysis (Qiao et al., 2015a). Figure 2 shows the Mn-*L* sXAS-TEY spectra collected on Na_0.44_MnO_2_ with different charging and discharging voltages (Figure 2A), which demonstrate the variation of Mn on the electrode surface. The experimental spectra (solid lines) of the Na_0.44_MnO_2_ are well reproduced (dotted lines) by quantification through linear combination of three reference spectra of Mn^2+^ (MnO), Mn^3+^ (Mn_2_O_3_), and Mn^4+^ (Li_2_MnO_3_). It is concluded from Figure 2 that (1) surface Mn is oxidized to higher oxidation states at high-voltage charge state and is reduced to lower oxidation states at low-voltage charge state; (2) a significant concentration of Mn^2+^ is formed on the surface of Na_*x*_MnO_2_ electrodes at low electrochemical potential. These spectroscopic results together with the electrochemical profiles upon extended cycling indicate that the surface Mn^2+^ is detrimental and responsible for capacity decay in the Na_0.44_MnO_2_ electrodes.

This finding provides the rationality to improve the electrochemical performance. By regulating the discharge cutoff voltage to above 3 V, the surface Mn^2+^ is greatly suppressed, leading to an enhanced cycling stability. A surface coating method is also suggested to be effective to suppress the surface Mn^2+^ formation and enhance the cycling life. This case demonstrates how the quantitative analysis based on TM-*L* sXAS-TEY benefits the understanding on the surface redox mechanisms and the rational optimization of a battery electrode material.

### Transition Metal Redox in Na*_*x*_*[MnFe(CN)_6_] With Interstitial Water Effect

**Figure 3:**
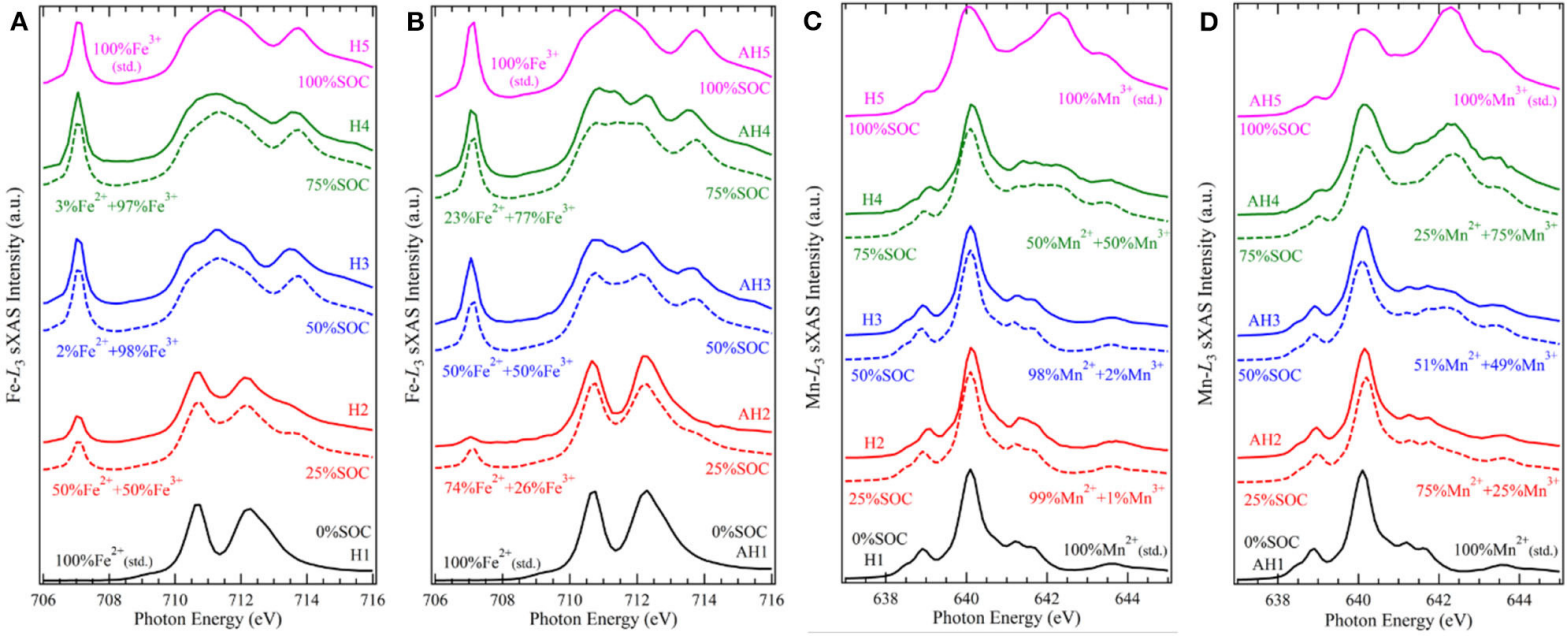
Fe and Mn *L*_3_-edge soft X-ray absorption spectroscopy (sXAS) spectra collected on a series electrode samples of Na_*x*_MnFe(CN)_6_·*z*H_2_O systems, **(A)** Fe for hydrated, **(B)** Fe for anhydrated, **(C)** Mn for hydrated, and **(D)** Mn for anhydrated. The calculated values were achieved by linear fitting of reference spectra. Reprinted from Wu et al. (2017) with no permission required from authors.

The coordination environments could often strongly affect the electrochemical profile via affecting the electronic states of the redox centers. Hexacyanometallates, i.e., Prussian blue analogs (PBAs), are a series of promising NIB cathode candidates for both aqueous and electrolytes, due to their ease of synthesis, low cost, and rigid open framework with large interstitial space for sodium ions. Song *et al*. find that the interstitial H_2_O molecule has a strong effect on the cycling performance of Na_*x*_[MnFe(CN)_6_]; i.e., with the interstitial H_2_O removed from the material, the electrochemical behavior is greatly improved, changing from a two-plateau to single-plateau profile with much reduced polarization, but the mechanism remains ambiguous (Song et al., 2015). To illustrate the mechanisms of this effect, we conduct TM-*L* sXAS measurements on the Na_*x*_[MnFe(CN)_6_] samples at different states of charge, and we compare the spectroscopic results for the hydrated and anhydrated sets of samples (Wu et al., 2017). Figure 3 displays the Fe (A, B) and Mn (C, D) *L*-edge sXAS-TFY spectra both collected by experiments and calculated by linear fitting of reference spectra. It can be seen that (1) in the hydrated system, the Fe^2+/3+^ and Mn^2+/3+^ redox reactions occur respectively at the low potential and high potential voltage plateaus, leading to a two-plateau cycling profile; (2) in the anhydrated system, Fe^2+/3+^ and Mn^2+/3+^ redox reactions take place spontaneously throughout the electrochemical process in a single cycling plateau.

The different TM redox sequences in the comparative systems are attributed to the competing effect between the ligand field stabilization energy (LFSE) and the standard ionization energy of TMs. In the conventional wisdom, the TM redox potential depends on the ionization energy under particular oxidation states. As the standard ionization energy of Fe^2+^ is lower than that of Mn^2+^, the standard electrode potential of Fe^2+^ is usually lower than that of Mn^2+^. Further, in the Na_*x*_[MnFe(CN)_6_] system, Fe and Mn are coordinated with (C=N)^−^ and (N=C)^−^, defining their spin states as LS and HS, respectively. The LFSE of LS Fe^2+^ is higher than that of HS Mn^2+^, leading to the increase of Fe^2+/3+^ redox potential. The above two factors, i.e., standard ionization potential and LFSE, have an opposite effect on the cycling potential, leading to a mixed redox process and a single plateau in the anhydrated samples. However, the balance of the competing effect between the LFSE with specific spin states and the ionization energy is easily broken by the change of structure and/or crystal field strength of the materials. The interstial H_2_O molecule, a seemingly subtle role, carries a big weight, by diluting the ligand field in the FeC_6_ and MnN_6_ octahedra and disturbing the original structure that defines the spin states. This makes the potential gap of the conventional Fe^2+/3+^ and Mn^2+/3+^ redox to re-emerge and results to the two-plateau in the hydrated system. This work showcases the precise quantification capability of TM-*L* sXAS on 3*d* TM redox and indicates the unique opportunity for optimizing the electrochemical performance by interstitial molecules.

### Fingerprint of Bulk Mn Redox in Na_0.6_Li_0.2_Mn_0.8_O_2_ by Transition Metal-*L* Mapping of Resonant Inelastic X-Ray Scattering

TM-*L* sXAS plays a unique role to quantitatively analyze the redox activities both on the surface and in the bulk via the two modes TEY and TFY that have different probe depths; however, in some scenarios, the TFY mode may become ineffective. For example, in Mn oxide compounds, which are a series of commonly used battery electrode materials, Mn-*L* sXAS-TFY will present a distorted lineshape due to the self-absorption and saturation effects. Mn-*L* mRIXS-iPFY has been clarified a bulk probe without distortion (Achkar et al., 2011) and is utilized to study the bulk Mn redox as a perfect alternative to sXAS-TFY (Dai et al., 2019; Ji et al., 2020; Wu et al., 2020a,b).

**Figure 4:**
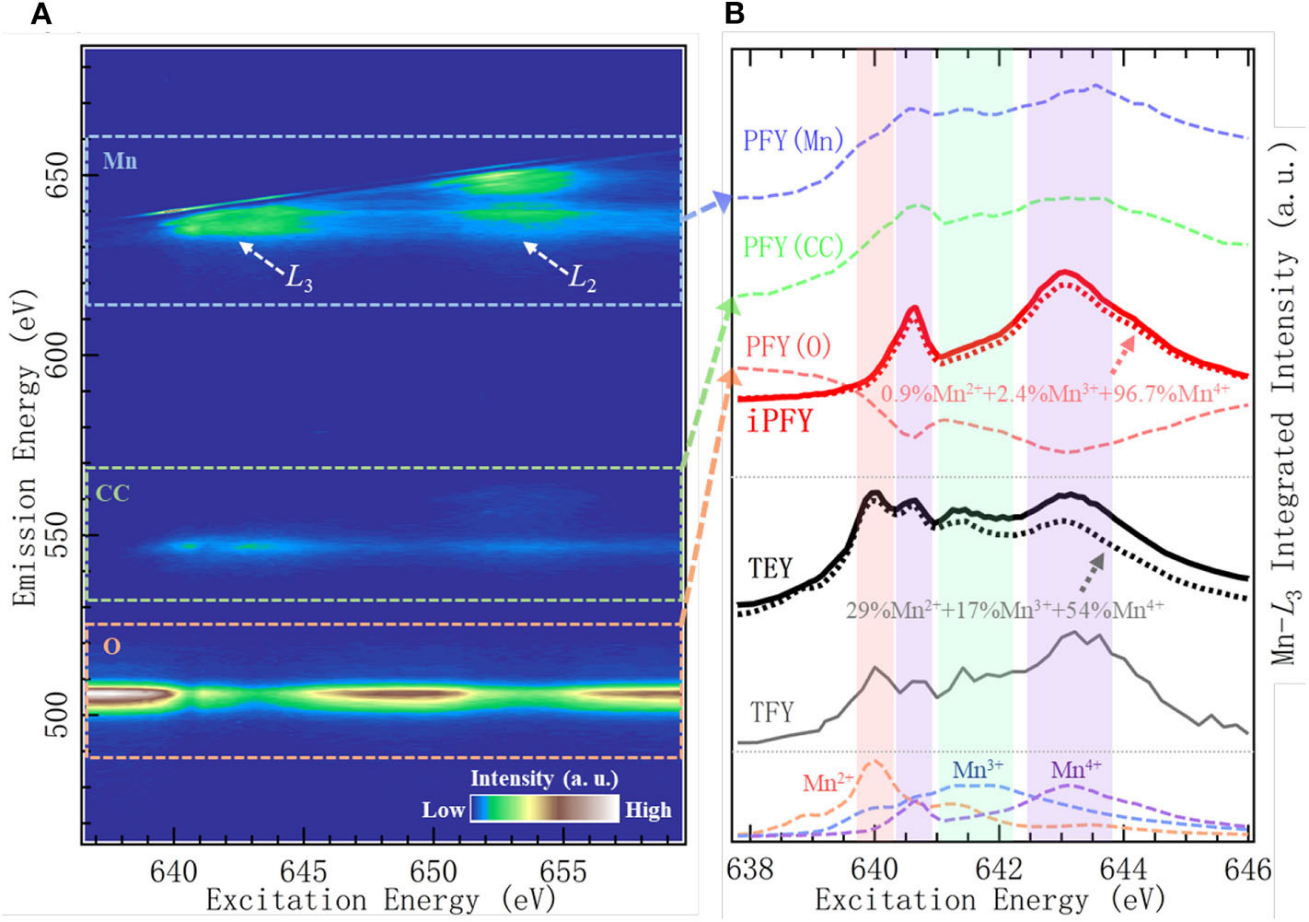
Mn-*L* mapping of resonant inelastic X-ray scattering (mRIXS) **(A)** and various absorption profiles **(B)** of Na_0.6_Li_0.2_Mn_0.8_O_2_. Reproduced from Wu et al. (2020b) with no permission required from authors.

Figures 4A,B show the Mn-*L* mRIXS and several absorption profiles of the Na_0.6_Li_0.2_Mn_0.8_O_2_ electrode at one specific state of charge, i.e., first discharged to 3.95 V (other information regarding this material to be detailed in *Dissociation of Lattice O Redox and Performance Decay in Na-Ion Battery Cathodes*) (Wu et al., 2020b). In Figure 4A, the normalized intensity is characterized by the color scale on the map as a function of both excitation and emission energies. It can be seen that *L*_2_- and *L*_3_-edge are well separated along with the excitation energy; meanwhile, by virtue of high-emission energy resolution, mRIXS is split into three parts along the emission energy axis, corresponding to three different decay processes, i.e., Mn-*L* 3*d*-2*p* edge (between 620 and 660 eV within dashed blue rectangle), Mn core-core 3*s*-2*p* edge (between 530 and 570 eV within dashed green rectangle), and O-*K* 2*p*-1*s* edge (between 510 and 525 eV within orange rectangle) (Golnak et al., 2016; Yang and Devereaux, 2018). By integrating the signal intensity within each part, three different partial FYs (PFY) are respectively achieved as PFY(Mn), PFY(CC), and PFY(O), shown in Figure 4B. iPFY is calculated through the formula iPFY = *a*/PFY(O), where *a* is a normalization coefficient. TEY and TFY spectra are measured from conventional Mn-*L* sXAS, and in principle, TFY is also equivalent to the sum of the three PFYs. It can be seen from Figure 4B that, while the surface probe TEY has a well-performed lineshape, the bulk probe TFY is seriously distorted and thus not reliable; in the meantime, both PFY(Mn) and PFY (CC) encounter broadened distortion due to the saturation effect, and only iPFY is the reliable bulk probe and feasible for quantification. The dotted black and red lines are the curve fitting results via linear combination with the standard experimental spectra of Mn^2+/3+/4+^, as listed in the bottom of Figure 4B. The quantification results demonstrate that iPFY is dominant by Mn^4+^, and TEY presents plenty of Mn^2+/3+^. This contrast between iPFY and TEY clearly indicates the different redox reactions on the surface from that in the bulk. To be more specific, in the Na_0.6_Li_0.2_Mn_0.8_O_2_ system, low-valence Mn^2+^ on the surface behaves in a counterintuitive pattern; i.e., it increases significantly during the charging (oxidation) process and reaches the maximum value at the fully charged state (Wu et al., 2020b), suggesting significant surface reactions taking place during the high-potential charge involving electrolyte degradation (Qiao et al., 2015b).

Based on this accurate quantification of bulk Mn, the sophisticated coupling relationship between Mn redox and O redox can be illuminated in this representative NIB system with O redox, as elaborated later in *Dissociation of Lattice O Redox and Performance Decay in Na-Ion Battery Cathodes*.

### Novel Mn(I) State in Na*_*x*_*Mn[Mn^II^(CN)_6_]_0.81_

**Figure 5:**
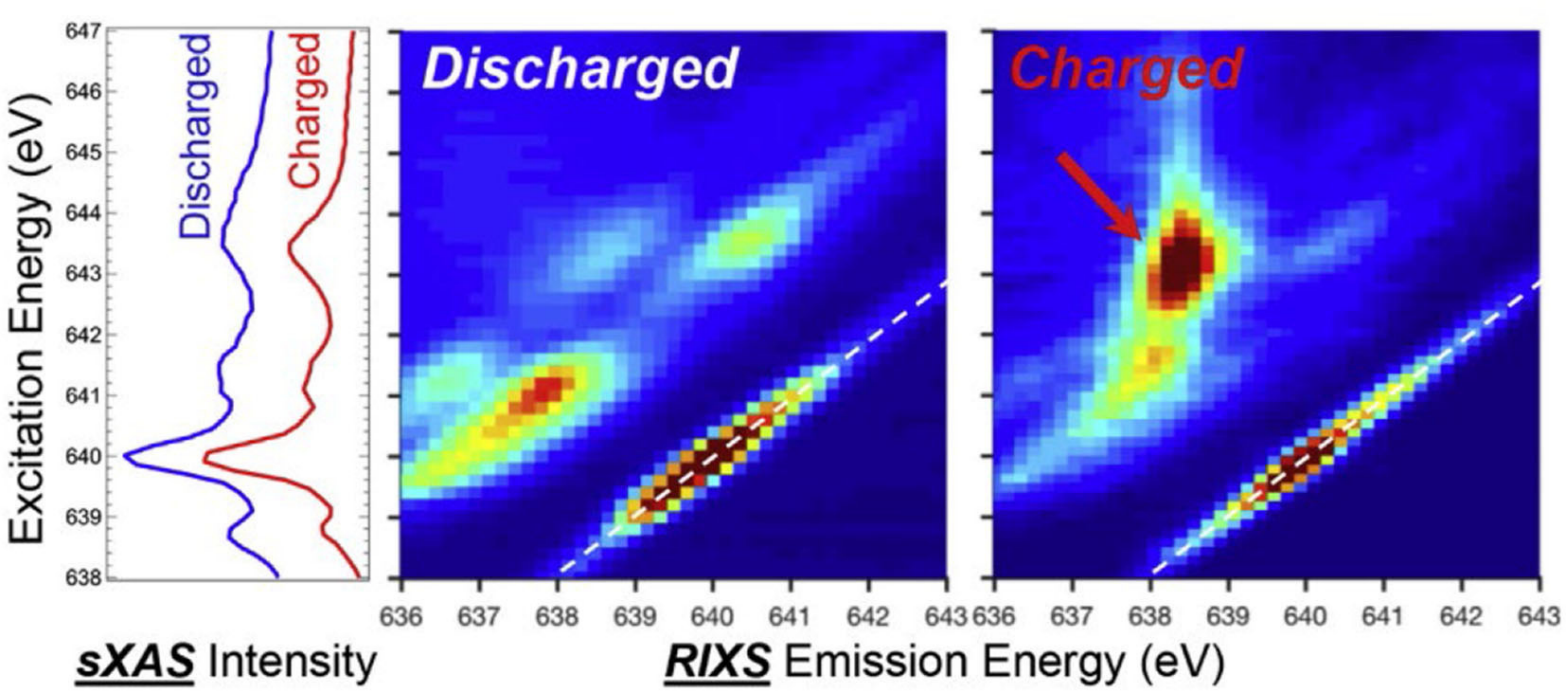
Soft X-ray absorption spectroscopy (sXAS) and mapping of resonant inelastic X-ray scattering (mRIXS) data collected on charged and discharged anode material Na_2.05_Mn[Mn^I^(CN)_6_]_0.81_ and Na_1.24_Mn[Mn^II^(CN)_6_]_0.81_. Reprinted from Yang and Devereaux (2018) with permission from Elsevier.

Compared with sXAS, mRIXS provides new dimension information to resolve the entangled states in sXAS, by measuring the emission photon energy distribution at each excitation energy. This offers an incisive method to determine the novel TM redox couples in battery materials, for instance, Mn(I/II) redox. Figure 5 shows the sXAS and mRIXS data collected on charged and discharged anode materials Na_2.05_Mn[Mn^I^(CN)_6_]_0.81_ and Na_1.24_Mn[Mn^II^(CN)_6_]_0.81_ (Firouzi et al., 2018). This PBA material can be used as a high-rate NIB anode with an intriguing low-voltage plateau. Researchers propose that the low-voltage plateau responds to Mn(I/II) redox; however, this cannot be proved by the conventional sXAS. Although in this PBA material sXAS is thought to be reliable to probe Mn states due to the well-defined spin state depending on their coordination sites, there is only a negligible difference around 643.5 eV observed for the charged and discharged electrodes at the left panel of Figure 5. In sharp contrast, the mRIXS results reveal distinct difference between the Mn^1+^ and Mn^2+^ samples at the middle and left panels of Figure 5. The discharged Mn^2+^ sample displays several groups of the *d*-*d* excitation features (parallel the elastic peak) with partially occupied *t*_2g_ and *e*_g_ states. The charged Mn^1+^ sample displays an enhanced RIXS feature (red arrow) with a high energy-loss value at the excitation energy of 643.5 eV. This distinct feature of charged sample corresponds to the *d*-*d* excitation of a low-spin Mn^1+^ 3*d*^6^ system with fully occupied *t*_2g_ states. In this way, mRIXS provides the direct experimental verification of the Mn(I/II) redox in Na-ion anode electrode. This case demonstrates the limitation of the sXAS and the power of mRIXS for revealing novel TM chemistry and suggests a promising application prospect of mRIXS in battery community.

## O Redox in Sodium-Ion Battery Cathodes

O redox is a novel conceptual breakthrough that has been progressing rapidly during the past couple of years. Though proposed firstly in the Li-rich compounds, this concept has also been found in the NIB cathode materials and has become a promising strategy to enhance the capacity. In this section, we summarize several advancements of the O redox mechanisms in the NIB cathodes. In the very beginning, we discuss on the characterization methods of the O electronic states in the battery electrodes.

### Detecting Lattice O Redox States Through Mapping of Resonant Inelastic X-Ray Scattering

To decipher the O redox mechanisms, a direct and reliable spectroscopic probe of the intrinsic O electronic states is essential. While O-*K* sXAS has been popularly employed in the studies of O redox (Oishi et al., 2015, 2016; Luo et al., 2016a,b; Ma et al., 2017), it has been clarified that the pre-edge feature evolution in both the intensity and lineshape is largely determined by the TM states, which is hard to be distinguished from the oxygen redox states (Yang and Devereaux, 2018; Roychoudhury et al., 2020). In principle, mRIXS has an additional ability of resolving the fluorescence along the emission energy and thus is capable to disentangle the two features (Qiao et al., 2017). Therefore, O-*K* mRIXS is a more creditable spectroscopic tool on the O redox issues.

**Figure 6:**
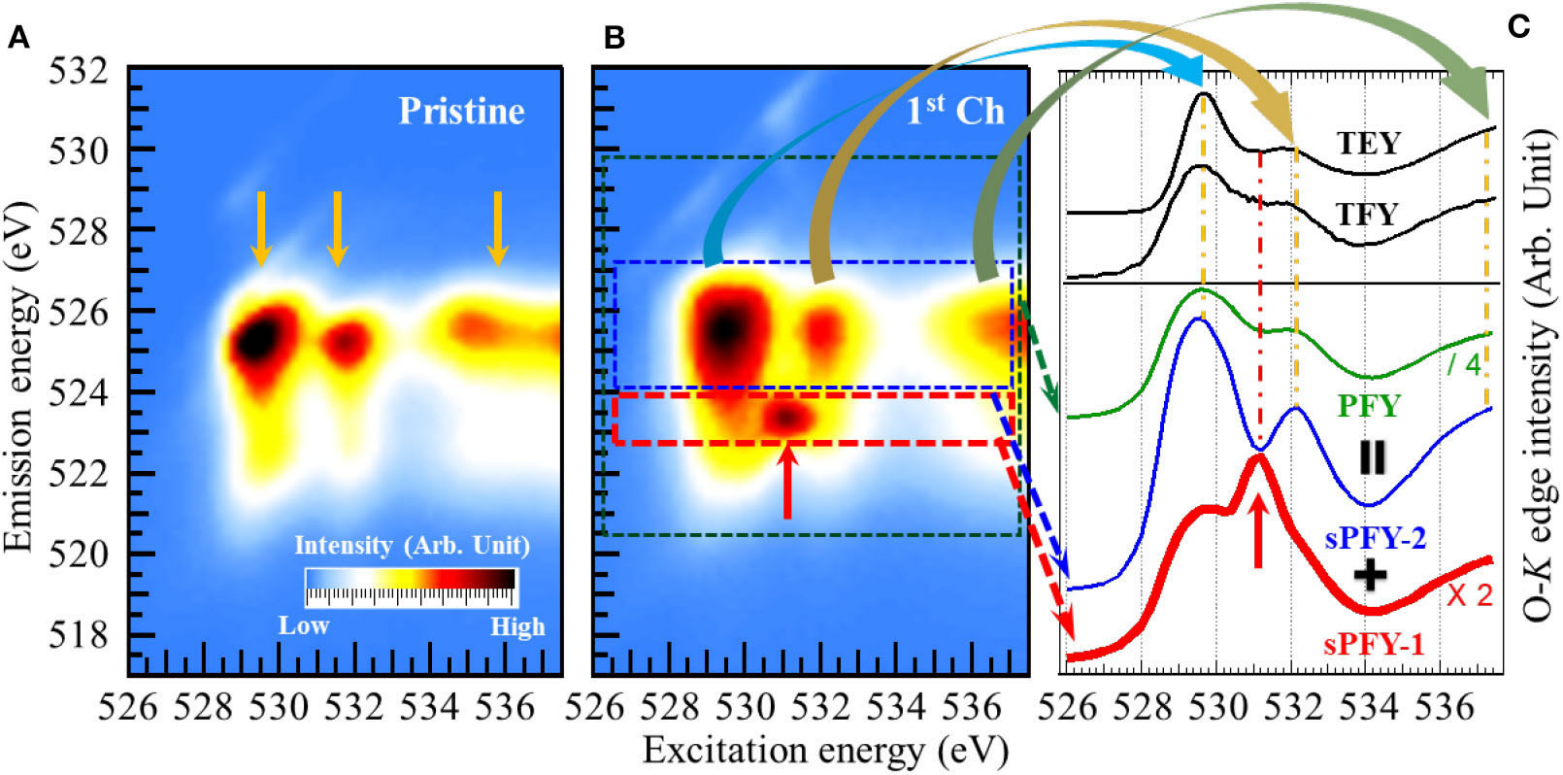
Mapping of resonant inelastic X-ray scattering (mRIXS) of pristine **(A)** and first charged **(B)**, and various absorption profiles **(C)** of Na_2/3_Mg_1/3_Mn_2/3_O_2_. A distinct oxidized O feature (indicated by the red arrow) emerges around 531.0-eV excitation energy and 523.7-eV emission energy on the O-*K* mRIXS of the charged electrodes. While this signature feature is buried under the lineshapes of all the conventional absorption profiles total electron yield (TEY), total fluorescence yield (TFY), and partial fluorescence yield (PFY), it becomes outstanding on the super-partial fluorescence yield (sPFY)-1. Reprinted from Dai et al. (2019) with permission from Elsevier.

To be more specific, we make a comparison between O-*K* sXAS and mRIXS via a model NIB cathode Na_2/3_Mg_1/3_Mn_2/3_O_2_, which is the first NIB system explored by mRIXS (other information regarding this material to be detailed in *Reversibility and Cyclability of O Redox in Na-Ion Battery Cathodes*) (Dai et al., 2019). In the study, it is observed from Figures 6A,B that a distinct feature around 531.0-eV excitation energy and 523.7-eV emission energy on the O-*K* mRIXS appears on the first charged electrode, as indicated by the red arrow. The feature is identical with the charged Li-rich NMC sample Li_1.17_Ni_0.21_Co_0.08_Mn_0.54_O_2_ (Gent et al., 2017) and acknowledged as the signature of the oxidized O species in the lattice of the battery electrodes. It is noted that this feature was also observed on the O-*K* mRIXS of the other non-divalent compounds such as O_2_ gas and Li_2_O_2_, with the same excitation energy around 531.0 eV but different shapes (Zhuo et al., 2018, 2020), indicating that the appearance of this signature feature is material-dependent.

However, this distinct RIXS feature of oxidized O species becomes merged on the conventional sXAS. Figure 6C demonstrates several absorption profiles of the first charged Na_2/3_Mg_1/3_Mn_2/3_O_2_ electrode. The two black solid lines in the upper panel are TEY and TFY spectra achieved from O-*K* sXAS measurement. Integrating the intensity within the main O-*K* signal range (520- to 530-eV emission energy as indicated by the green dash rectangle) gives the conventional PFY. It can be seen that none of the TEY, TFY, and PFY lineshapes presents any obvious feature around 531.0-eV excitation energy. Actually, aside from the signature feature of the oxidized O species, there are three other intensity packets (indicated by the yellow arrows) on the O-*K* mRIXS, which have been identified as the RIXS features from O-2*p* band (above 534-eV excitation energy) and its hybridizations with TMs (529- and 532-eV excitation energy, namely, pre-edge region), as reviewed previously (Butorin et al., 2000; Wu et al., 2019). The three features dominate the lineshapes of the conventional absorption profiles and overwhelm the oxidized O feature underneath the pre-edge region. This explains the deficiency of sXAS and the advantage of mRIXS on probing the signature feature of lattice oxidized O species.

To differentiate the oxidized O feature and TM-O hybridization feature, two new absorption profiles were proposed, namely, super-PFY (sPFY), by integrating the intensity within the red and blue dashed rectangles. While sPFY-2 shows a consistent lineshape with the conventional sXAS, sPFY-1 makes the signature feature of the oxidized O species outstanding as indicated by the red arrow. Therefore, sPFY-1 can be a reliable and quantifiable alternative to characterize O redox in battery electrodes (hereafter, sPFY is specifically referred to as sPFY-1).

### Reversibility and Cyclability of O Redox in Na-Ion Battery Cathodes

As we mentioned above, O redox is a promising strategy to enhance the capacity of the battery electrodes, but only highly reversible and cyclable O redox is meaningful in terms of practical application. In this sense, a dependable quantitative evaluation on the reversibility and cyclability of O redox is essential. Here, we clarify that the “reversibility” is indicated by the ratio between the discharge and charge capacity contributions from the lattice O redox, and the “cyclability” refers to the ratio between the extended and first discharge capacity contributions from the lattice O redox. Before O-*K* mRIXS was utilized in the studies of NIB cathodes, many researches have claimed “reversible” O redox; unfortunately, they are not conclusive due to the unreliability of the spectroscopic techniques such as O-*K* XPS and sXAS, as we discussed above.

**Figure 7:**
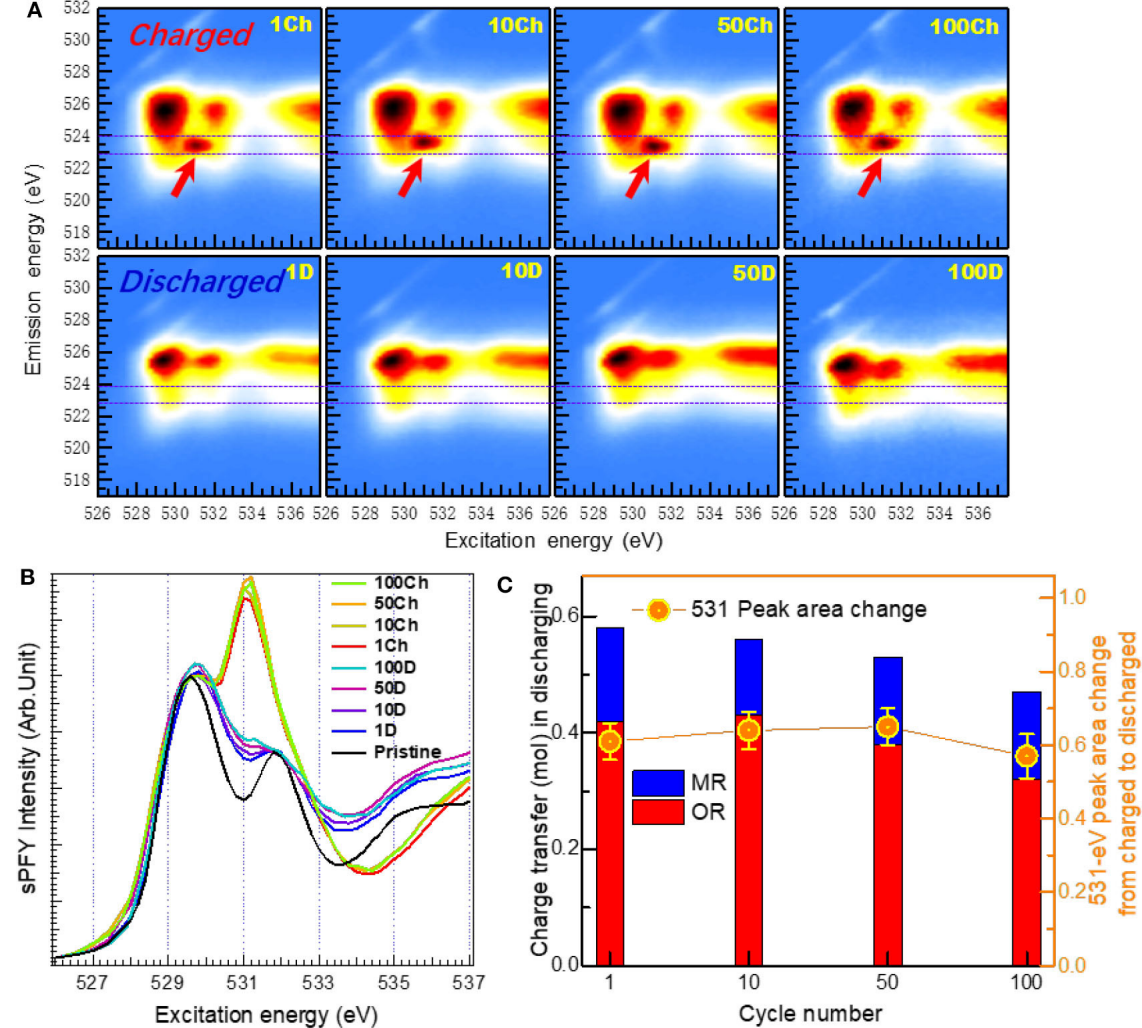
Evaluation of reversibility and cyclability of O redox upon extended cycles. **(A)** O-*K* mapping of resonant inelastic X-ray scattering (mRIXS) and **(B)** super-partial fluorescence yield (sPFY) of charged and discharged electrodes after the 1st, 10th, 50th, and 100th cycles. **(C)** Quantification of the capacity contribution from O and Mn redox in the unit of charge transfer. Reprinted from Dai et al. (2019) with permission from Elsevier.

Dai *et al*. establish a quantification method based on O-*K* mRIXS-sPFY to quantitatively evaluate the reversibility and cyclability of the O redox in Na_2/3_Mg_1/3_Mn_2/3_O_2_ (Dai et al., 2019). With this deliberately designed stoichiometry, this material has pure high-valence Mn^4+^ in the pristine state (i.e., nominally no low-valence Mn^2+/3+^) and presents high initial charge capacity, making itself a perfect candidate to study the evolution activity of O redox. Figure 7 demonstrates the O-*K* mRIXS and sPFY of the Na_2/3_Mg_1/3_Mn_2/3_O_2_ electrodes. A distinct feature around 531.0-eV excitation energy on the O-*K* mRIXS-sPFY varies upon the electrochemical cycling, i.e., enhances as a hump during charging and weakens as a dip during discharging. This feature actually corresponds to the signature packet of the lattice oxidized O species on the O-*K* mRIXS. By a simple integration of the sPFY intensity around 531.0 eV (e.g., from 530.2 to 532 eV for Na_2/3_Mg_1/3_Mn_2/3_O_2_), the area variation between different states of charging is achieved, which can be referred to as a spectroscopic indicator to evaluate the lattice O redox activities in the electrodes. In this way, the reversibility and cyclability can be quantified. It is worth noting that the sPFY spectra have been scaled to the peak of the TM-O hybridization feature (e.g., 529.7 eV for Na_2/3_Mg_1/3_Mn_2/3_O_2_) before the integration, and different spectral normalization does not change the specific value of the reversibility and cyclability, because only contrast of the area is of concern. According to the quantification results in Figure 7, it is concluded that the reversibility of the lattice O redox during the initial cycle is 79%, and the cyclability over 100 cycles is 87%; i.e., both the reversibility and cyclability are quite high in the model NIB system. To verify the universality of the quantification method, it is also utilized on the Li-rich NMC compounds Li_1.17_Ni_0.21_Co_0.08_Mn_0.54_O_2_. As a comparison, Li_1.17_Ni_0.21_Co_0.08_Mn_0.54_O_2_ has an initial reversibility of 76% and a cyclability of 44% over the 500th cycle.

Via a combined analysis based on Mn-*L* mRIXS-iPFY and O-*K* mRIXS-sPFY, the capacity contributions from the cationic and anionic redox are quantified respectively in the battery cathode, and some critical findings regarding the O redox mechanisms are further discussed. Firstly, with extended cycles for Na_2/3_Mg_1/3_Mn_2/3_O_2_, it can be found that the high-voltage plateau disappears after only 10 cycles. This suggests that the lattice O redox could take place at low voltage, and it is not valid to assume the high-voltage plateau as the indicator of O redox. The dissociation between the high-voltage plateau and O redox is also observed in Li-rich compounds (Gent et al., 2017). Secondly, during the high-voltage plateau of the initial cycle, the area variation on the O-*K* mRIXS-sPFY of Li_1.17_Ni_0.21_Co_0.08_Mn_0.54_O_2_ is much lower than that of Na_2/3_Mg_1/3_Mn_2/3_O_2_, with the same electrochemical charge compensation. This finding indicated that, while all capacity at high-voltage plateau of Na_2/3_Mg_1/3_Mn_2/3_O_2_ stems from lattice O redox, the non-lattice O reactions, e.g., the O_2_ gas release or other surface reactions, contribute to the capacity during the high-voltage plateau of Li_1.17_Ni_0.21_Co_0.08_Mn_0.54_O_2_.

### Dissociation of Lattice O Redox and Performance Decay in Na-Ion Battery Cathodes

Because Na_2/3_Mg_1/3_Mn_2/3_O_2_ contains almost pure lattice O redox in, it is indeed a very distinctive cathode system, as surface or non-lattice O activities (e.g., the O_2_ gas release or other surface reactions) commonly exist in many battery cathodes. Considering the consistency with the lattice O redox, we call these O activities as non-lattice O “redox,” even though only irreversible O “oxidization” is involved. In conventional wisdom, the O redox activities, without differentiating either lattice or non-lattice O redox, are generally believed to be detrimental to the electrochemical performance. But whether this judgment is correct for lattice O redox still remains elusive and has become a critical issue.

**Figure 8:**
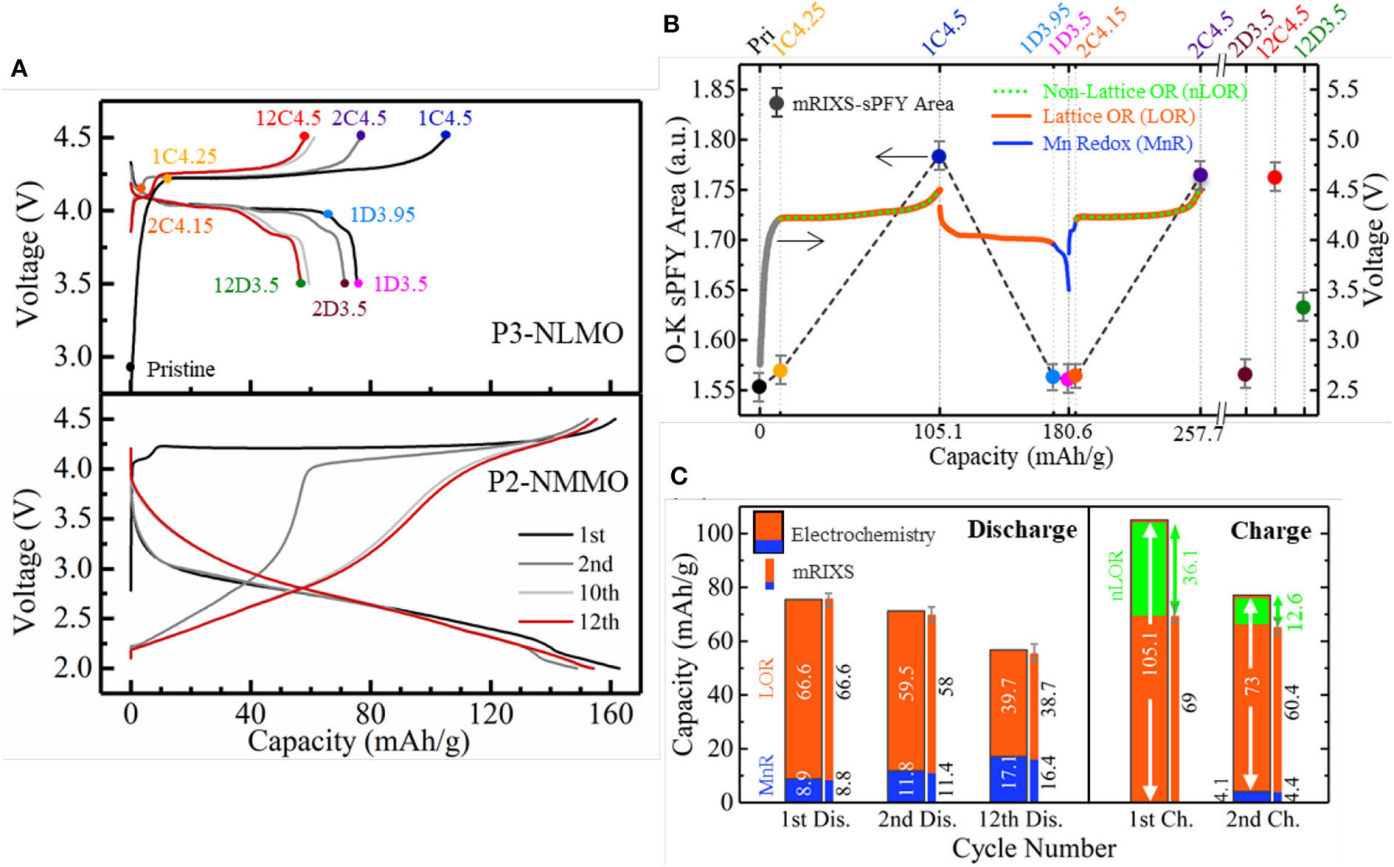
A comparative study on Na_0.6_Li_0.2_Mn_0.8_O_2_ (NLMO) and Na_2/3_Mg_1/3_Mn_2/3_O_2_ (NMMO). **(A)** Cycling profiles of NLMO and NMMO. **(B)** Differentiation of three redox reactions in NLMO during cycling. **(C)** Capacity contributions from three redox activities that are quantified based on electrochemical capacity (wide bar) and mapping of resonant inelastic X-ray scattering (mRIXS) results (thin bar). Reprinted from Wu et al. (2020b) with no permission required from authors.

As the lattice and non-lattice O redox usually couples together in the battery cathodes during cycling, it is essential to distinguish them first so that the individual effect of the two O activities can be clarified separately. We propose an analytical scheme that made this feasible within the model NIB cathode Na_0.6_Li_0.2_Mn_0.8_O_2_ (Wu et al., 2020b). Due to the nominal high-valence Mn^4+^ in pristine material with Li doping, it can be inferred that a notable amount of O redox activities is involved in the cycling of Na_0.6_Li_0.2_Mn_0.8_O_2_, similar with Na_2/3_Mg_1/3_Mn_2/3_O_2_. While Na_2/3_Mg_1/3_Mn_2/3_O_2_ is a rather unique system with almost pure lattice O redox (Dai et al., 2019), Na_0.6_Li_0.2_Mn_0.8_O_2_ is actually an analog candidate that potentially contains both lattice and non-lattice O redox and thus provides an excellent opportunity for comparative and detailed investigation. As shown in Figure 8A, the NLMO electrode shows an obvious voltage drop and a growing low-voltage discharge plateau with only tens of cycles. This triggers an intriguing question that whether the O redox leads to these performance decays in such a system with dominant O redox.

By utilizing the O-*K* mRIXS-sPFY and TM-*L* mRIXS-iPFY on the model, the lattice O redox and TM redox are quantified in terms of capacity contributions, and the non-lattice O redox is consequentially invoked as the gap between the total electrochemical capacity and the capacity contributions from the TM/O redox. To this point, the three different redox activities, i.e., TM redox and lattice and non-lattice O redox, are decoupled quantitatively, as shown in Figures 8B,C. Several conclusions can be suggested. Firstly, the low-voltage plateau reaction is due to Mn^3+/4+^ redox, and the huge capacity decay during the initial cycles mainly resulted from the non-lattice O redox, even though the emerging Mn redox contribution cannot compensate the lost non-lattice O redox capacity. Secondly, the growing low-voltage plateau during discharge is due to the continuously increasing Mn^3+/4+^ redox upon cycling. This means that the lattice O redox itself is not the culprit of the performance decay. This is an important correction to the conventional wisdom and suggests that the lattice O redox should be treated separately with other O activities so that viable O redox-based electrodes for high-performance batteries could be achieved.

### O Redox With Negligible Voltage Hysteresis in Na-Ion Battery Cathodes

Another critical issue for the O redox system is the strong voltage hysteresis and the sluggish kinetics, which has been described as the most important practical issue for utilizing O redox (Assat and Tarascon, 2018). For the Li-ion battery electrodes, either the Li-rich or non-Li-rich conventional compounds have strong voltage hysteresis. In contrast with the Li-rich systems, NIB electrodes can realize reversible O redox without excessive Na-ion; e.g., doping Li/Mg or introducing vacancy into the TM layer could also trigger the reversible O redox. However, these compounds present either distinct voltage hysteresis (Dai et al., 2019) or pretty low retainability due to the irreversible O activities (Wu et al., 2020b), making themselves not feasible for practical utilization.

**Figure 9:**
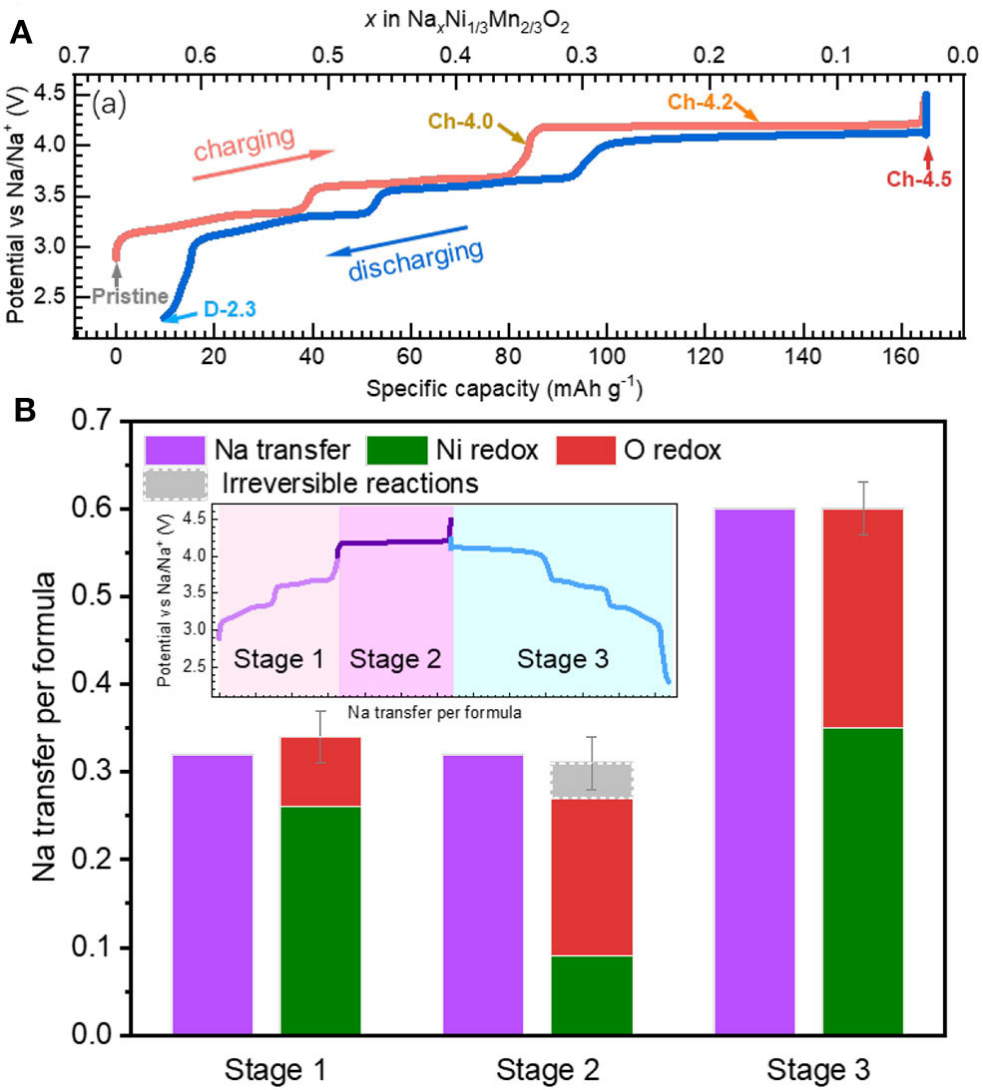
Electrochemistry and redox activities in Na_2/3_Ni_1/3_Mn_2/3_O_2_ (NNMO). **(A)** Electrochemical profile of NNMO with negligible voltage hysteresis. **(B)** Quantitative evaluation on the electrochemical capacity from TM and O redox. Reprinted from Dai et al. (2020) with permission from *Nano Energy*.

Recently, Dai et al. revisit a conventional 3*d*-TM oxide, i.e., Na_2/3_Ni_1/3_Mn_2/3_O_2_, that displays several striking properties (Dai et al., 2020). Firstly, it has only a voltage hysteresis of about 0.1 V, as shown in Figure 9A. This is very low compared with Li-rich or some of the NIB electrodes, e.g., Na_2/3_Mg_1/3_Mn_2/3_O_2_. Secondly, unlike other NIB electrodes, this compound presents a highly reversible electrochemical profile with well-defined plateaus close to each other during initial cycling, leading to a relatively high Columbic efficiency. Thirdly, the compound is highly air-stable and has an excellent rate and cycling performance, indicating great practical potentials and facile kinetics (Mao et al., 2019).

Here triggers a critical question whether O redox or TM redox leads to such excellent electrochemical behaviors of low-voltage hysteresis, high reversibility, and high-rate properties. Dai *et al*. differentiate the redox activities during the initial cycle based on TM-*L* sXAS and O-K mRIXS, as shown in Figure 9B. It is clarified that lattice O redox activities take place during the electrochemical cycling of Na_2/3_Ni_1/3_Mn_2/3_O_2_ on both the low- and high-voltage plateaus. This indicates that the lattice O redox could lead to a highly reversible electrochemical profile with a relatively low-voltage hysteresis and highly reversible profile lineshape, which is usually taken as an inherent feature of the TM redox.

Scientifically, it is worth noting that Na_2/3_Ni_1/3_Mn_2/3_O_2_ consists only of 3*d*-TM elements in the TM-O layer. While the Li/Mg doped NIB electrodes, e.g., Na_0.6_Li_0.2_Mn_0.8_O_2_ and Na_2/3_Mg_1/3_Mn_2/3_O_2_, include ionic bonding Li/Mg-O, similar with Li-rich compounds, the Na_2/3_Ni_1/3_Mn_2/3_O_2_ is a true non-alkali-rich system. This indicates that the lattice O redox does not require the alkali-rich coordination environment. Meanwhile, the Ni redox could nominally compensate all the charge transfer during cycling and the reversible O redox could still take place, without the exhaustion of TM redox. Moreover, the O redox and TM redox are coupled together from low-voltage stage to high-voltage stage. This contrasts to most other systems with O redox such as Li-rich or Li/Mg doped Na-ion electrodes, in which O and TM redox occur separately during the initial charging.

## Summary and Perspectives

In this review, we summarize the advancements of the cationic and anionic redox mechanisms in the NIB electrodes that have been deciphered by SXS. Through TM-*L* sXAS and mRIXS-iPFY, the valence states of TM-3*d* could be quantitatively characterized both on the surface and in the bulk of NIB electrodes. The ability of resolving the emitted photon energy in mRIXS greatly enhances the chemical sensitivity beyond conventional sXAS experiments, making mRIXS the “tool of choice” for probing some particular TM and O states in battery electrodes, such as the monovalent Mn in anodes and non-divalent O in cathodes with O redox reactions.

We discuss several examples of the TM redox mechanisms in NIB cathode materials that have been revealed by sXAS and mRIXS. For the oxide-based compound Na_0.44_MnO_2_, the Mn-ion concentrations on the surface are quantified by Mn-*L* sXAS-TEY, verifying the formation of the critical surface Mn^2+^ species. For the hexacyanometallate Na_*x*_MnFe(CN)_6_, the variations of bulk Fe and Mn upon cycling are both quantitatively fingerprinted, illustrating different Fe/Mn redox sequences in the hydrated and anhydrated systems. mRIXS-iPFY provides a non-distorted bulk probe of Mn, which enables the quantifications of the bulk Mn redox in the Na_0.6_Li_0.2_Mn_0.8_O_2_ cathode. More strikingly, in the case of Na_*x*_Mn[Mn(CN)_6_]_0.81_, the existence of novel monovalent Mn is revealed directly by Mn-*L* mRIXS, showcasing the superior chemical sensitivity of mRIXS on unveiling novel states in batteries.

For the O redox in the NIB cathode materials, we elaborate that mRIXS is a reliable probe of the lattice O redox activities in battery electrodes. mRIXS-sPFY analysis provides quantitative information on the reversibility and cyclability of the lattice O redox by following the oxidized O feature intensity variation upon electrochemical cycling. Several interesting findings regarding the O redox activities in the NIB cathodes have been reported in terms of several critical discussions on the reversibility and cyclability, electrochemical performance decay, and voltage hysteresis and sluggish kinetics. It is concluded by the case of Na_2/3_Mg_1/3_Mn_2/3_O_2_ that the lattice O redox can be highly reversible and retainable in the NIB cathodes, verifying the potential for practical utilization of this promising strategy. A comparative study on Na_0.6_Li_0.2_Mn_0.8_O_2_ and Na_2/3_Mg_1/3_Mn_2/3_O_2_ shows that the reversible lattice O redox is not the culprit of the capacity and voltage decay and should be treated separately with the irreversible O redox. While O redox systems almost always display sluggish kinetic, indicated by a large voltage hysteresis, mRIXS studies of Na_2/3_Ni_1/3_Mn_2/3_O_2_ found that the system with strong O redox, however, facile kinetics and highly reversible electrochemical profile, directly challenges our conventional wisdom and triggers future studies on the root cause of the kinetics problem of O redox reactions.

Fundamentally, the O redox mechanisms in NIB electrodes remain ambiguous, and critical scientific questions remain elusive (Assat and Tarascon, 2018; Yang, 2018). The most profound challenge is the intrinsic nature of the oxidized O species in battery electrodes, which could be finally resolved through the theoretical interpretation of the distinct O-redox signature in O-*K* mRIXS. This remains a grand challenge to the fields of physics, chemistry, and material sciences at this time but is critical for understanding and controlling lattice O redox.

We note that SXS is still evolving to meet the need of today's energy material researches with its full potential that is yet to be explored. We believe this review on SXS of NIB redox mechanism will encourage more studies on both the technical developments and scientific discoveries. Recent mRIXS studies found that the technique is sensitive to subtle chemical changes of O affected by inductive effects or by solvation shell configurations (Jeyachandran et al., 2014; Wu et al., 2020a). This indicates that mRIXS could be used for detailed studies of polyanionic NIB systems. Furthermore, with the new generation of diffraction limited light sources and further RIXS spectrometer upgrades into the spatial and temporal domains (Chuang et al., 2020), mRIXS will become more and more powerful and enable new opportunities for in-depth analysis of NIB materials.

## Author Contributions

WY and ZS conceived this work. JW and WY wrote the manuscript. All authors reviewed and contributed to the discussions.

## Conflict of Interest

The authors declare that the research was conducted in the absence of any commercial or financial relationships that could be construed as a potential conflict of interest.
